# Evolution of anthozoan polyp retraction mechanisms: convergent functional morphology and evolutionary allometry of the marginal musculature in order Zoanthidea (Cnidaria: Anthozoa: Hexacorallia)

**DOI:** 10.1186/s12862-015-0406-1

**Published:** 2015-06-30

**Authors:** Timothy D. Swain, Jennifer L. Schellinger, Anna M. Strimaitis, Kim E. Reuter

**Affiliations:** Department of Civil and Environmental Engineering, Northwestern University, Evanston, IL 60208 USA; Department of Zoology, Field Museum of Natural History, Chicago, IL 60605 USA; Department of Biological Science, Florida State University, Tallahassee, FL 32306-4295 USA; Department of Biology, Temple University, Philadelphia, PA 19122 USA

**Keywords:** Phylogenetic comparative methods, Functional morphology, Convergent evolution, Evolutionary allometry, Coelenterata, Hexacorallia, Symbiosis

## Abstract

**Background:**

Retraction is among the most important basic behaviors of anthozoan Cnidaria polyps and is achieved through the coordinated contraction of at least six different muscle groups. Across the Anthozoa, these muscles range from unrecognizable atrophies to massive hypertrophies, producing a wide diversity of retraction abilities and functional morphologies. The marginal musculature is often the single largest component of the retraction mechanism and is composed of a diversity of muscular, attachment, and structural features. Although the arrangements of these features have defined the higher taxonomy of Zoanthidea for more than 100 years, a decade of inferring phylogenies from nucleotide sequences has demonstrated fundamental misconceptions of their evolution.

**Results:**

Here we expand the diversity of known marginal muscle forms from two to at least ten basic states and reconstruct the evolution of its functional morphology across the most comprehensive molecular phylogeny available. We demonstrate that the evolution of these forms follows a series of transitions that are much more complex than previously hypothesized and converge on similar forms multiple times. Evolution of the marginal musculature and its attachment and support structures are partially scaled according to variation in polyp and muscle size, but also vary through evolutionary allometry.

**Conclusions:**

Although the retraction mechanisms are diverse and their evolutionary histories complex, their morphologies are largely reflective of the evolutionary relationships among Zoanthidea higher taxa and may offer a key feature for integrative systematics. The convergence on similar forms across multiple linages of Zoanthidea mirrors the evolution of the marginal musculature in another anthozoan order (Actiniaria). The marginal musculature varies through evolutionary allometry of functional morphologies in response to requirements for additional force and resistance, and the specific ecological and symbiotic functions of individual taxa.

**Electronic supplementary material:**

The online version of this article (doi:10.1186/s12862-015-0406-1) contains supplementary material, which is available to authorized users.

## Background

Expansion and retraction are the most basic and important behaviors of cnidarian polyps. Anthozoans expand their columns and tentacles for prey capture and handling [1–4], sediment removal [5, 6], fending-off predators [7] and competitors [8], exposure of photosynthetic symbionts (when localized in the tentacles, pseudotentacles, or column vesicles) to light [4, 9–11], and to increase oxygen and waste product diffusion (including removal of excess oxygen created by symbionts that can reduce metabolic rates and cause cellular damage from hyperoxia [12, 13]) across an increased surface area [14]. Anthozoans retract their columns and tentacles to avoid detection by predators [4, 9], escape predation (in the startle response), prevent desiccation [15], protect photosynthetic symbionts from intense irradiance or ultraviolet radiation [4, 14], reduce metabolic rates [9, 10] and generate low internal oxygen tension [16, 17], and reduce oxygen and waste product diffusion (potentially concentrating superoxide radicals created by symbiont photosynthesis under hyperoxic conditions [12, 13]) across a reduced surface area [4].

Expansion is achieved through the action of cilia lining the siphonoglyph(s) of the actinopharynx that pump water into the coelenteron and contiguous tentacles to create hydrostatic pressure [15]. Expansion is maintained by closing the opening to the actinopharynx and additional ciliary action to sustain hydrostatic pressure [10]. Retraction is achieved by release of pressure (allowing water to escape from the coelenteron) and contraction of circular muscles lining the column walls and longitudinal muscles of the mesenteries [15]. Both behaviors require energetic expenditure (beating of cilia and contraction of muscles to close the mouth, or contraction of relatively large circular and longitudinal muscles); however, because retraction simultaneously creates demand for (through muscle contraction) and reduces the source of (surface area) oxygen, the energetic cost to retraction is thought to be greater [18] and unsustainable [4]. As structures are generally retracted when their primary functions (prey capture, light collection, etc.) are impeded, their expansion must involve some additional cost (e.g. exposure to damage) that makes retraction energetically justifiable [9, 10].

The retraction mechanisms of Anthozoa includes circular columnar (and tentacular) and marginal muscles, longitudinal mesenteric retractor and parietal muscles, and oblique mesenteric parietobasilar muscles [15]. These muscles are differentially developed among Anthozoa taxa ranging from apparent absences to massive hypertorphies. Because the forces that muscles are able to generate are proportional to their cross-sectional area, the extent of muscle development is an indication of the capabilities and necessities of each taxon. When present, the marginal muscles work (as a drawstring) in conjunction with the retractor muscles (that depress the oral disk) to cover the retracted oral disk and tentacles with the margin of the column and provide an important defense from predators and desiccation [15, 19]. Species with highly developed marginal muscles are often intertidal or live in exposed, wave-swept habitats [20, 21]; those with underdeveloped or absent marginal muscles are often greatly reduced in size or have shifted to an infaunal habitat [22].

The marginal musculature of Hexacorallia (also known as the marginal sphincter or Rotteken’s muscle of Actiniaria) arises through hypertrophy of columnar circular muscles at the margin of the polyp [19, 23, 24]. The columnar circular muscles, which line the length of the column, are often anchored to miniscule mesogleal pleats (increasing surface area for muscle attachment). To support the hypertrophied marginal musculature, these pleats become enlarged, merge together to partially enclose the muscle within the mesoglea, or completely envelope the muscle within the mesoglea [23, 25]. In some species where hypertrophied marginal muscles are absent, the disk and tentacles remain exposed during contraction; suggesting that the structure of the marginal musculature can determine the extent of retraction ability for the polyp [26].

The evolutionary history of the marginal musculature of Hexacorallia is complex, with multiple independent origins of the hypertrophy and reversals to the unenlarged state [24, 27, 28]. The marginal musculature is best studied in the Actiniaria, but is well known in Zoanthidea and less so in Corallimorpharia and Scleractinia (the diffuse endodermal musculature of Scleractinia receives little attention as the taxonomy and systematics of this order are based on skeletal structure [29–31]); it is apparently absent in Antipatharia and Ceriantharia [24, 28]. Extant taxa exhibit marginal muscles that are generally characterized as absent (atrophy), endodermal (muscles are anchored to the gastrodermal face of the mesoglea), transitional (muscles are anchored to the gastrodermal face of the mesoglea and partially embedded within the mesoglea) or mesogleal (muscles are entirely embedded within the mesoglea). Endodermal and mesogleal marginal musculatures are synapomorphic in Actiniaria [24, 27, 28], and were (until recently) thought be the same in Zoanthidea [32]. Because of the origin and development of the marginal musculature, Zoanthidea exhibiting the endodermal form were considered most basal and those exhibiting the double mesogleal form considered most derived [19, 23], while transitional forms were considered to be a demonstration of the “transference” from endodermal to mesogloeal form within a single species [23, 25]. A recent molecular analysis [28] recovered two independent origins of the marginal musculature of Actiniaria (both mesogleal) and a third in Zoanthidea (again mesogleal, although a more comprehensive analysis was inconclusive [32]).

Given the functional importance of the marginal musculature and long-standing hypotheses of its evolution, it is a logical extension that these structures should be important to systematists. Even a cursory review of the Zoanthidea (and Actiniaria) taxonomic literature reveals particular interest in the mesogleal structures that support the marginal musculature; although impossible to visualize without the application of hydrofluoric acid and preparation-intensive histological sectioning [33, 34], a drawing or photograph of the marginal mesoglea is often the only image included in species descriptions. These historical images and illustrations provide a record of rich diversity of form, however this diversity was generally partitioned in systematics as a simple binary character (mesogleal or endodermal) with few variants (transitional or double) or qualifiers (diffuse or concentrated). Ectodermal and mesogleal marginal musculature states have defined Zoanthidea families and genera for more than 100 years, however this binary character is now known to be homoplasious [32] and its application (as a definitive binary character) to systematics is untenable.

Here we provide an assessment of the diversity and evolution of Zoanthidea contraction mechanisms by reconstructing the functional morphology of the marginal musculature across the most comprehensive molecular phylogeny available. The results indicate that there are at least ten recognizable forms of extant marginal musculature, whose evolutionary history are much more complex (converging on mesogleal or endodermal forms at least 5 times) than previously recognized. Throughout the evolutionary history of Zoanthidea marginal musculature, size of the muscles vary over two orders of magnitude and shape of the mesogleal support structures mirror that variation allometrically. Despite this complexity, the arrangement of the mesogleal structures supporting the marginal muscles display low levels of homoplasy and appear to be synapomorphic for multiple clades of species recognizable through molecular and ecological characters; restoring the relevance of the marginal musculature as a defining character in Zoanthidea systematics.

## Results

### Diversity and descriptions of extant marginal muscle forms

**Fig. 1:**
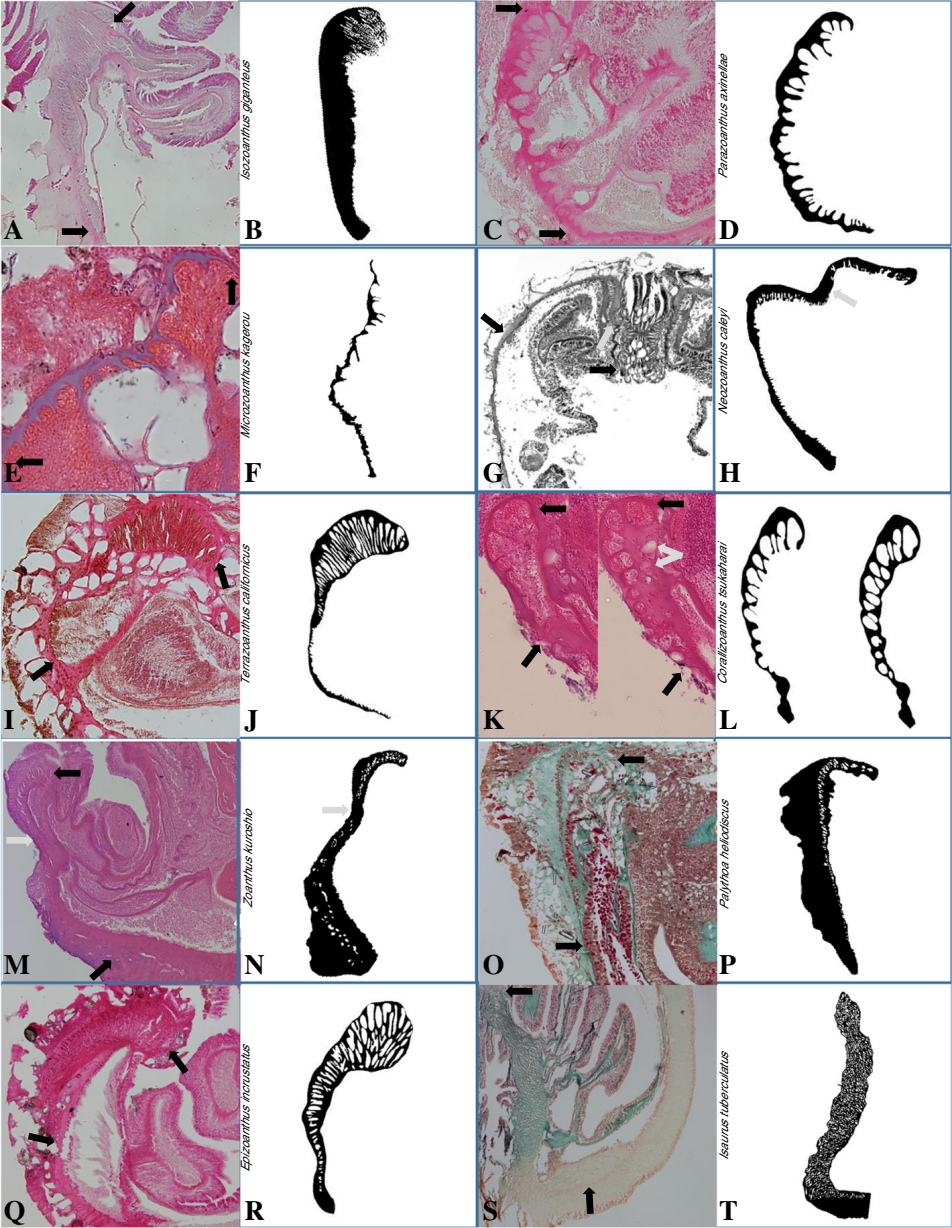
Marginal musculature arrangements. Photographs of histological section of marginal musculature (between black arrows) with accompanying drawing of isolated mesogleal structures supporting the marginal musculature for branchiform endodermal (*Isozoanthus giganteus*: **a**, **b**), cteniform endodermal (*Parazoanthus* axinellae: **c**, **d**), spindly-cteniform endodermal (*Microzoanthus kagerou*: **e**, **f**), discontiguous endodermal (*Neozoanthus caleyi*: **g**, **h**; grey arrow indicates undifferentiated mesoglea; histological image reproduced with permission of J. Reimer), meso-endo transitional (*Terrazoanthus californicus*: **i**, **j**), cyclically transitional (*Corallizoanthus tsukaharai*: **k**, **l**; grey arrows indicate lacunae formed by dissolution of foraminifera), discontiguous mesogleal (*Zoanthus kuroshio*: **m**, **n**; grey arrow indicates undifferentiated mesoglea), linear mesogleal (*Palythoa heliodiscus*: **o**, **p**), reticulate mesogleal (*Epizoanthus incrustatus*: **q**, **r**), orthogonally-reticulate mesogleal (*Isaurus tuberculatus*: **s**, **t**)

A review of histological sections and compiled literature on taxa represented in the composite phylogeny, revealed a minimum of ten distinct marginal musculature arrangements (Fig. 1). The two main historical character states, endodermal and mesogleal, are divided into seven arrangements (i.e. novel character states): branchiform endodermal, cteniform endodermal, spindly-cteniform endodermal, discontiguous endodermal, linear mesogleal, reticulate mesogleal, and orthogonally-reticulate mesogleal. The two primary historical variations, divided (or double) mesogleal and transitional, are directly translated to discontiguous mesogleal and divided into cyclically transitional (*sensu* Swain & Swain [34]), and meso-endo transitional forms, respectively.

**Branchiform endodermal** arrangements, as seen in longitudinal section of the polyp, have long gill-like mesogleal pleats supporting the distal 5–20 % of the length of the marginal musculature, transitioning abruptly to a cteniform-like morphology with relatively short and uniform mesogleal pleats through the remaining length (Fig. 1a–b; Morphbank collection 851143; dimensions in Table 1). The branchiform section is composed of 28–58 $\left(\overline{\mathrm{x}} = 44,\ {\mathrm{n}}_{\mathrm{sections}} = 20\right)$ mesogleal pleats distally, dramatically reducing in pleat-length proximally, with the cteniform section composed of 117–220 $\left(\ \overline{\mathrm{x}} = 171,\ {\mathrm{n}}_{\mathrm{sections}} = 20\right)$ mesogleal pleats (Fig. 1a–b).

**Table 1:** Summary of marginal musculature dimensions within each character state

Muscle form	n_sections_	Length (μm)	Width (μm)	# of supports	Size of supports (μm)	Muscle CS-area (x 10^4^ μm^2^)
Branchiform endodermal	20	2796–4623 $\left(\overline{\mathrm{x}} = 3723\right)$	394–843 $\left(\overline{\mathrm{x}} = 564\right)$	151–275 $\left(\overline{\mathrm{x}} = 214\right)$	114–541 $\left(\overline{\mathrm{x}} = 256\right)$	23.6–40.9 $\left(\overline{\mathrm{x}} = 29.8\right)$
Cteniform endodermal	156	175–1251 $\left(\overline{\mathrm{x}} = 534\right)$	29–291 $\left(\overline{\mathrm{x}} = 77\right)$	7–79 $\left(\overline{\mathrm{x}} = 32\right)$	9–90 $\left(\overline{\mathrm{x}} = 32\right)$	0.1–4.2 $\left(\overline{\mathrm{x}} = 1.09\right)$
Spindly-cteniform endodermal	10	268–331 $\left(\overline{\mathrm{x}} = 298\right)$	34–65 $\left(\overline{\mathrm{x}} = 52\right)$	10–16 $\left(\overline{\mathrm{x}} = 15\right)$	15–36 $\left(\overline{\mathrm{x}} = 27\right)$	0.3–0.5 $\left(\overline{\mathrm{x}} = 0.4\right)$
Discontiguous endodermal	2	820–1049 $\left(\overline{\mathrm{x}} = 935\right)$	68–78 $\left(\overline{\mathrm{x}} = 73\right)$	46–60 $\left(\overline{\mathrm{x}} = 53\right)$	34–49 $\left(\overline{\mathrm{x}} = 41\right)$	0.7–2.4 $\left(\overline{\mathrm{x}} = 1.57\right)$
Meso-endo transitional	36	756–1536 $\left(\overline{\mathrm{x}} = 1023\right)$	114–263 $\left(\overline{\mathrm{x}} = 168\right)$	47–110 $\left(\overline{\mathrm{x}} = 71\right)$	77–200 $\left(\overline{\mathrm{x}} = 119\right)$	1.3–5.2 $\left(\overline{\mathrm{x}} = 2.77\right)$
Cyclically transitional	40	307–793 $\left(\overline{\mathrm{x}} = 483\right)$	57–137 $\left(\overline{\mathrm{x}} = 89\right)$	9–42 $\left(\overline{\mathrm{x}} = 23\right)$	22–72 $\left(\overline{\mathrm{x}} = 47\right)$	0.5–2.0 $\left(\overline{\mathrm{x}} = 9.25\right)$
Discontiguous mesogleal	10	1111–1324 $\left(\overline{\mathrm{x}} = 1192\right)$	153–271 $\left(\overline{\mathrm{x}} = 224\right)$	130–184 $\left(\overline{\mathrm{x}} = 151\right)$	32–76 $\left(\overline{\mathrm{x}} = 54\right)$	1.3–2.0 $\left(\overline{\mathrm{x}} = 1.73\right)$
Linear mesogleal	19	1219–3028 $\left(\overline{\mathrm{x}} = 1982\right)$	86–419 $\left(\overline{\mathrm{x}} = 273\right)$	69–136 $\left(\overline{\mathrm{x}} = 110\right)$	25–107 $\left(\overline{\mathrm{x}} = 66\right)$	1.3–12.9 $\left(\overline{\mathrm{x}} = 5.0\right)$
Reticulate mesogleal	53	884–2080 $\left(\overline{\mathrm{x}} = 1474\right)$	132–445 $\left(\overline{\mathrm{x}} = 246\right)$	51–298 $\left(\overline{\mathrm{x}} = 125\right)$	56–339 $\left(\overline{\mathrm{x}} = 153\right)$	3.55–17.4 $\left(\overline{\mathrm{x}} = 8.50\right)$
Orthogonally-reticulate esogleal	6	3652–4205 $\left(\overline{\mathrm{x}} = 3892\right)$	264–350 $\left(\overline{\mathrm{x}} = 314\right)$	628–834 $\left(\overline{\mathrm{x}} = 740\right)$	31–65 $\left(\overline{\mathrm{x}} = 46\right)$	44.2–48.2 $\left(\overline{\mathrm{x}} = 4.65\right)$

**Cteniform endodermal** arrangements, as seen in longitudinal section of the polyp, have uniformly short comb-like mesogleal pleats supporting the entire length of the marginal musculature on a lunate mesogleal base (Fig. 1c–d; Morphbank collection 851272; dimensions in Table 1).

**Spindly-cteniform endodermal** arrangements, as seen in longitudinal section of the polyp, have diminishingly short comb-like mesogleal pleats concentrated at the distal end of the marginal musculature on a sigmate mesogleal base (Fig. 1e–f; Morphbank collection 851278; dimensions in Table 1). The spindly-cteniform endodermal form differs from the cteniform endodermal form in that mesoglea supporting the marginal musculature is only known to be thin, sigmate, and sparsely populated by widely spaced pleats which become shorter proximally, with an overall appearance that suggests frailness. Only a single specimen from a single species was available for this form, therefore the form and its dimensions should be considered preliminary.

**Discontiguous endodermal** arrangements, as seen in longitudinal section of the polyp, have a distal aggregation of pleats, transitioning through a distinct sigmate region containing undifferentiated mesoglea (i.e. lacking pleats; gray arrow in Fig. 1g–h), ending in a proximal aggregation of pleats attached to a lunate mesogleal base (Fig. 1g–h; dimensions in Table 1). The two aggregations of pleats are asymmetrical, with the distal aggregation composed of notably fewer pleats (12–16, $\overline{\mathrm{x}} = 14,\ {\mathrm{n}}_{\mathrm{sections}} = 2$) than the proximal aggregation (34–44, $\overline{\mathrm{x}} = 56,\ {\mathrm{n}}_{\mathrm{sections}} = 2$; Fig. 1g–h). The discontiguous endodermal form differs from the cteniform endodermal form in that mesoglea supporting the marginal musculature is distinctly sigmate, with separate distal and proximal aggregations of pleats divided by a region containing undifferentiated mesoglea. Only a single histological section (through two regions of marginal muscle; Fig. 1g) of this form is available, therefore the form and its dimensions should be considered preliminary.

**Meso-endo transitional** arrangements, as seen in longitudinal section of the polyp, have elliptical or lachrymiform lacunae organized in a stack (reminiscent of a section through the stacks of cisternae) distally and transition, through a distinct constriction and sigmate-curve, to mesogleal pleats proximally (Fig. 1i–j; Morphbank collection 851276; dimensions in Table 1). Approximately half the length of marginal muscle is enclosed within 25–88 $\left(\overline{\mathrm{x}} = 44,\ {\mathrm{n}}_{\mathrm{sections}} = 36\right)$ lacunae that occupy the full diameter of mesoglea distally, reducing in diameter prior to shifting toward endoderm proximally, with half of muscle attachment sites opening to the endoderm and forming 9–53 $\left(\overline{\mathrm{x}} = 27,\ {\mathrm{n}}_{\mathrm{sections}} = 36\right)$ mesogleal pleats (Fig. 1i–j). The meso-endo transitional form differs from the reticulate mesogleal form in the organization (stacked rather than haphazardly reticulate) and shape (elliptical rather than irregularly-shaped) of the lacunae and the consistent transition in attachment sites between lacunae to pleats through a sigmate curve in the mesoglea.

**Fig. 2:**
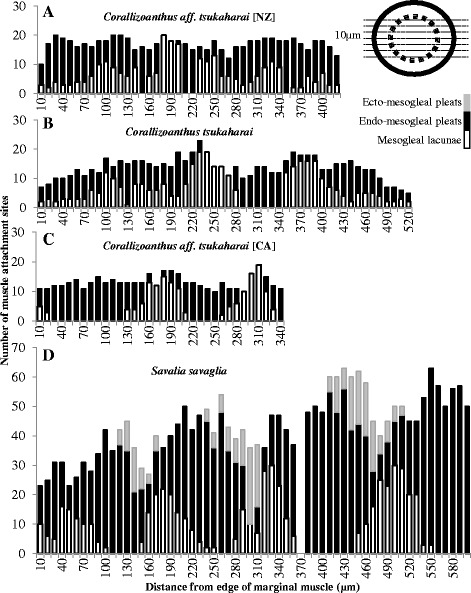
Patterns in the muscle attachment sites of cyclically transitional marginal muscle arrangements. Number, position, and type of marginal muscle attachment sites as they appear within serial longitudinal sections of *Corallizoanthus aff. tsukaharai* [NZ] (**a**), *Corallizoanthus tsukaharai* (**b**), *Corallizoanthus aff. tsukaharai* [CA] (**c**), and *Savalia savaglia* (**d**). Each bar represents a 10 μm longitudinal section with the number and type of muscle attachment points; gray bars indicate ectoderm-facing mesogleal pleats, black bars indicate endoderm-facing mesogleal pleats, open bars indicate mesogleal lacunae. Empty positions indicate data missing due to sectioning artifact. Inlay diagram demonstrates plane of microtome blade (dotted lines) against the diameter of the polyp (outer ring) and marginal muscle (broken ring)

**Cyclically transitional** arrangements (*sensu* Swain & Swain [34]), as seen in longitudinal section of the polyp, have muscle attachment sites that transition between sections from mesogleal pleats to mesogleal lacunae (Figs. 1k–l and 2a–d; Morphbank collection 851271; dimensions in Table 1), with a period of 20–220 μm $\left(\overline{\mathrm{x}} = 85,\ {\mathrm{n}}_{\mathrm{cycles}} = 23\right)$ per transition (Fig. 2a–d). Muscle fibers contained within 0–30 $\left(\overline{\mathrm{x}} = 8,\ {\mathrm{n}}_{\mathrm{sections}} = 254\right)$ subtly angular lacunae that occupy entire mesoglea distally; lacunae confined toward endoderm proximally, with proximal-most lacunae opening to endoderm and forming 0–63 $\left(\overline{\mathrm{x}} = 22,\ {\mathrm{n}}_{\mathrm{sections}} = 254\right)$ mesogleal pleats (Figs. 1k–l and 2a–d). In *Savalia savaglia*, the transition of attachment sites transverses the mesoglea such that the most distal muscle attachment sites are pleats arising from the ectodermal surface of the mesoglea and the muscle transits the entire width of the mesoglea within a single section (Fig. 2d). The second row of lacunae (near endoderm) in sections where muscle appears to be mesogleal are the result of dissolved encrustations (often foraminifera; grey arrow, Fig. 1k).

**Discontiguous mesogleal** arrangements, as seen in longitudinal section of the polyp, have a distal aggregation of circular or elliptical lacunae, transitioning through a distinct sigmate region containing unperforated mesoglea (i.e. lacking lacunae; grey arrow, Fig. 1m–n), ending in a proximal aggregation of lacunae embedded within the lunate mesogleal base (Fig. 1m–n; Morphbank collection 851280; dimensions in Table 1). The two aggregations of lacunae are usually asymmetrical, with the distal aggregation composed of notably fewer lacunae (32–76, $\overline{\mathrm{x}} = 54,\ {\mathrm{n}}_{\mathrm{sections}} = 10$) then the proximal aggregation (87–110, $\overline{\mathrm{x}} = 97,\ {\mathrm{n}}_{\mathrm{sections}} = 10$), and approximate a linear (single or multiple tracts) arrangement along the length of the marginal musculature (Fig. 1m–n). The discontiguous mesogleal form (which was historically categorized as the double sphincter muscle of Zoanthidea) is differentiated from all other mesogleal forms by the presence of distal and proximal concentrations of lacunae separated by unperforated mesoglea.

**Linear mesogleal** arrangements, as seen in longitudinal section of the polyp, have circular or elliptical lacunae that approximate a single, continuous, linear arrangement along the length of the marginal musculature (Fig. 1o–p; Morphbank collection 851279; dimensions in Table 1).

**Reticulate mesogleal** arrangements, as seen in longitudinal section of the polyp, have irregularly-shaped lacunae haphazardly arranged along the length of the marginal musculature such that the supporting mesoglea appears to be a reticulate mesh (Fig. 1q–r; Morphbank collection 851270; dimensions in Table 1). Mesogleal muscle occupies full diameter of mesoglea distally and often narrows near the proximal terminus (Fig. 1q–r). In some species the muscle may shift toward endoderm proximally, prior to transitioning to an endodermal tail, that ranges from half the length of the muscle to non-existent. Although a few species exhibiting the reticulate mesogleal form may have an endodermal tail (similar to the meso-endo transitional form), none are known to also have elliptical lacunae organized in a stack.

**Orthogonally-reticulate mesogleal** arrangements, as seen in longitudinal section of the polyp, have rectangular-shaped lacunae arranged in contoured grids along the length of the marginal musculature such that the supporting mesoglea appears to be an orthogonal mesh (Fig. 1s–t; Morphbank collection 851277; dimensions in Table 1). Mesogleal muscle occupies full diameter of mesoglea distally and often narrows and shifts toward endoderm near the proximal terminus (Fig. 1s–t).

Two additional arrangements, provisionally identified as simplified mesogleal and endo-meso transitional arrangements, are known only from published drawings or photographs, and may represent misinterpretations of other forms and require further examination to confirm if they represent true character states. The **simplified mesogleal** arrangement, as seen in published drawings of the longitudinal section of the polyp, have 7–17 circular or elliptical lacuna, arranged linearly, and may be an oversimplification of a known mesogleal or endodermal marginal muscle forms. **Endo-meso transitional** arrangements, as seen in the longitudinal section of the polyp, have ~28 linearly arranged attachment points that transition from endodermal (~50 % of points) to mesogleal (distal to proximal), and may be a misinterpretation of the cyclically transitional (as viewed in a single histological section) or cteniform endodermal arrangements. Due to the uncertainty surrounding the simplified mesogleal arrangement and its subsequent use in assignment of species to genera, we propose that *Epizoanthus cutressi* West is hereby reassigned to the genus *Parazoanthus* with the new binomen *Parazoanthus cutressi* (West), as indicated by molecular and ecological characters that demonstrate its affiliation with *Parazoanthus* [32, 35].

### Evolutionary relationships among extant forms

**Fig. 3:**
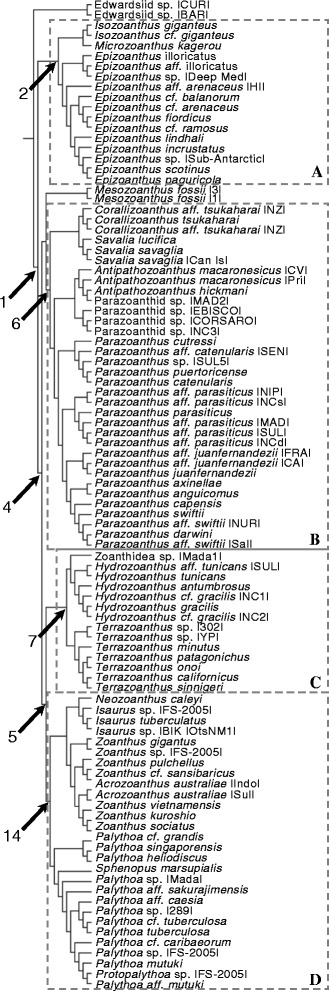
Composite evolutionary tree based on the topology of the molecular phylogeny of Swain [32] with additional taxa amended following the molecular analyses of Swain & Swain [34], Fujii & Reimer [37], and Reimer et al. [45]. Boxed regions show the area of the phylogeny detailed in Fig. 4 (**a**), Fig. 5 (**b**), Fig. 6 (**c**), and Fig. 7 (**d**)

**Fig. 4:**
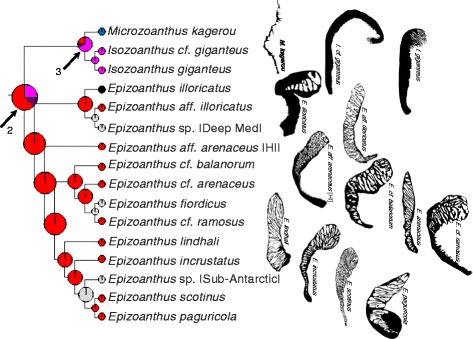
Maximum likelihood ancestral state reconstruction of marginal musculature form for the basal region of the composite evolutionary tree (Fig. 3a) populated by *Isozoanthus*, *Microzoanthus*, and *Epizoanthus* taxa. Drawings to the right of tree represent extant forms of the isolated mesogleal structures supporting the marginal musculature. Pie chart sections represent the relative likelihood of each character state (that exceeded 5 %) at the node and are enlarged at ancestral nodes to increase clarity

**Fig. 5:**
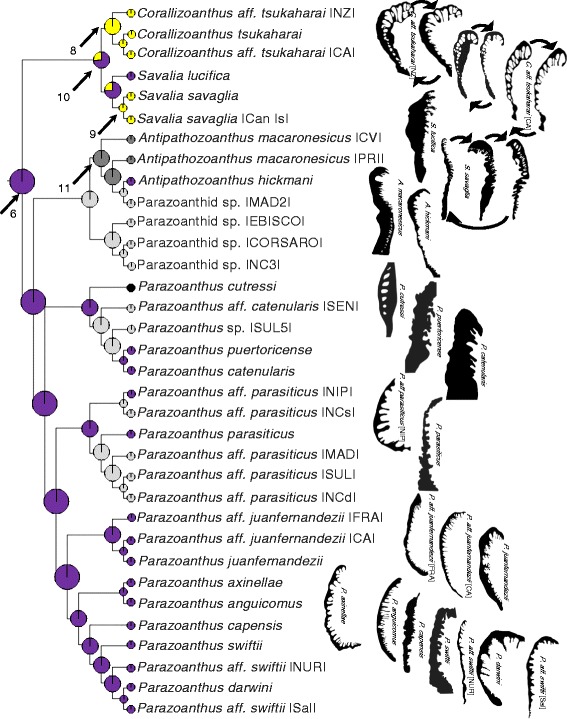
Maximum likelihood ancestral state reconstruction of marginal musculature form for the central region of the composite evolutionary tree (Fig. 3b) populated by *Corralizoanthus*, *Savalia*, *Antipathozoanthus*, and *Parazoanthus* taxa. Drawings to the right of tree represent extant forms of the isolated mesogleal structures supporting the marginal musculature. Pie chart sections represent the relative likelihood of each character state (that exceeded 5 %) at the node and are enlarged at ancestral nodes to increase clarity

**Fig. 6:**
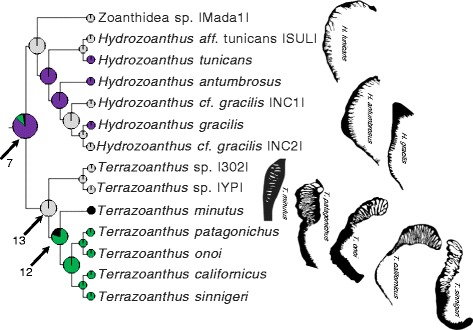
Maximum likelihood ancestral state reconstruction of marginal musculature form for the Hydrozoanthidae portion of the composite evolutionary tree (Fig. 3c) populated by *Hydrozoanthus* and *Terrazoanthus* taxa. Drawings to the right of tree represent extant forms of the isolated mesogleal structures supporting the marginal musculature. Pie chart sections represent the relative likelihood of each character state (that exceeded 5 %) at the node and are enlarged at ancestral nodes to increase clarity

**Fig. 7:**
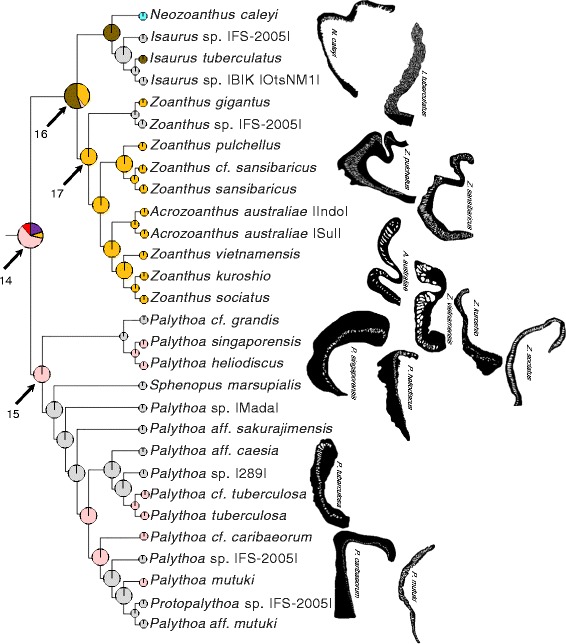
Maximum likelihood ancestral state reconstruction of marginal musculature form for the Brachycnemina portion of the composite evolutionary tree (Fig. 3d) populated by *Neozoanthus*, *Isaurus, Acrozoanthus*, *Zoanthus*, *Palythoa*, *Protopalythoa*, and *Sphenopus* taxa. Drawings to the right of tree represent extant forms of the isolated mesogleal structures supporting the marginal musculature. Pie chart sections represent the relative likelihood of each character state (that exceeded 5 %) at the node and are enlarged at ancestral nodes to increase clarity

The composite phylogeny (Fig. 3), an expansion of the topology of Swain [32], is the basis of all phylogenetic analyses presented here. Mapping the extant marginal muscle forms on the composite phylogeny, revealed similar forms among closely related taxa (Figs. 4, 5, 6, and 7). Of the ten primary forms, eight are exclusively identified from single genera (branchiform endodermal of *Isozoanthus*, spindly-cteniform endodermal of *Microzoanthus*, reticulate mesogleal of *Epizoanthus*, meso-endo transitional of *Terrazoanthus*, discontiguous endodermal of *Neozoanthus*, orthogonally-reticulate mesogleal of *Isaurus*, discontiguous mesogleal of *Zoanthus*, and linear mesogleal of *Palythoa*) and two are identified exclusively among closely related genera (cyclically transitional of *Corallizoanthus* and *Savalia*, and cteniform endodermal of *Antipathozoanthus*, *Parazoanthus*, and *Hydrozoanthus*; Figs. 4, 5, 6, and 7). The two provisional forms, endo-meso transitional and simplified mesogleal arrangements, are known respectively from a single species (*Antipathozoanthus macronesicus*) or three distantly related genera (*Epizoanthus* [formerly], *Parazoanthus*, and *Terrazoanthus*; Figs. 4, 5, and 6).

**Fig. 8:**
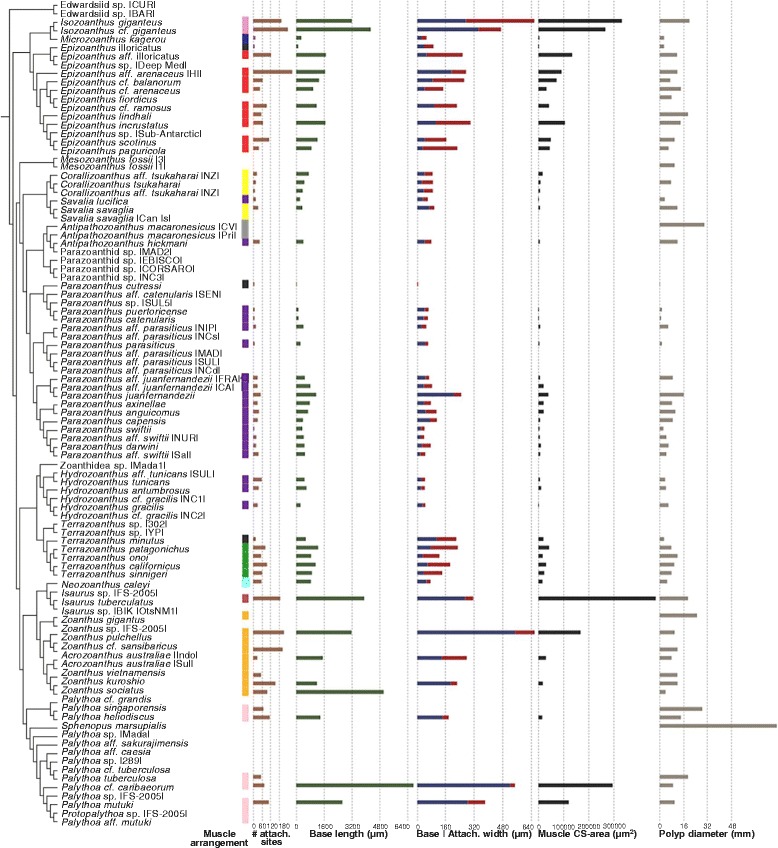
Marginal musculature arrangements and the dimensions of their structural components mapped to the composite phylogeny. Boxes indicate muscle arrangement character state (branchiform endodermal [purple], cteniform endodermal [violet], spindly-cteniform endodermal [blue], discontiguous endodermal [light blue], meso-endo transitional [green], cyclically transitional [yellow], discontiguous mesogleal [orange], linear mesogleal [pink], reticulate mesogleal [red], orthogonally-reticulate mesogleal [burgundy], endo-meso transitional [grey], simplified mesogleal [black]); bar graphs indicate the mean maximum number of attachment sites, mesogleal base length (μm), mesogleal base and attachment site width (μm), marginal muscle cross-sectional area (μm^2^), and polyp diameter (mm)

A similar pattern is seen among the shape and size parameters of the marginal musculature: similar shapes and sizes among closely related taxa, but great diversity across the entire phylogeny (Fig. 8). The genera that have the largest polyps, have the largest marginal muscle cross-sectional areas (*Isozoanthus*, *Epizoanthus*, *Isaurus*, *Zoanthu*s, and *Palythoa*), also have the most numerous (attachment site count) and robust support structures (base length, base mesoglea width, and attachment site width).

### Evolutionary history of the marginal musculature

The maximum likelihood (ML) ancestral state reconstructions recovered 9–15 transitions in the arrangement of the marginal musculature. The common ancestor of Zoanthidea (Fig. 3, node 1) most likely (proportional likelihood = 0.3329; all other states < 0.15) had a reticulate mesogleal marginal musculature (similar to extant *Epizoanthus*), which remained unchanged (proportional likelihood = 0.4433; Figs. 3 and 4, node 2) prior to the more recent shift to the branchiform endodermal arrangement (proportional likelihood = 0.4650; Fig. 4, node 3), followed by the transition to the autapomorphic spindly-cteniform endodermal (Fig. 4; *Microzoanthus kagerou*) arrangement. It is unclear if the marginal musculature of *Epizoanthus illoricatus* represents a state change or if the arrangement (known only from a drawing in the original description; Fig. 4) is an oversimplification of its true form.

An ancient transition from reticulate mesogleal to cteniform endodermal marginal musculature (similar to extant *Parazoanthus*, proportional likelihood = 0.9318; Fig. 3, node 4) remained unchanged in the common ancestor of the Hydrozoanthidae + Brachycnemina clade (proportional likelihood = 0.7959, all other states < 0.07; Fig. 3, node 5), the common ancestor of Parazoanthidae (proportional likelihood = 0.9613; Figs. 3 and 5, node 6), and the common ancestor of Hydrozoanthidae (proportional likelihood = 0.8343; Figs. 3 and 6, node 7). Two apparently independent transitions to the cyclically transitional arrangement are reconstructed at nodes 8 and 9, but a single transition to this state could be inferred earlier in evolutionary history (at node 10, Fig. 5) if the specimen representing *Savalia lucifica* in the molecular phylogeny has been misidentified and is actually a species with a cyclically transitional marginal musculature (see Discussion). Similarly, it is unclear if the marginal musculature of *Antipathozoanthus macaronesicus* (provisionally referred to as endo-meso transitional) represents a state change (Fig. 5; transition reconstructed at node 11 followed by a reversal in *Antipathozoanthus hickmani*) or if the arrangement (known only from a photograph of a single histological section in the original description) represents a misinterpretation of its true form. It is also unclear if the marginal musculature of *Parazoanthus cutressi* (provisionally referred to as simplified mesogleal) represents a state change or if the arrangement (known only from a drawing in the original description; Fig. 5) represents an oversimplification of its true form. A transition to the meso-endo transitional arrangement is reconstructed at node 12 (proportional likelihood = 0.7868; Fig. 6), but would be inferred to have occurred earlier in evolutionary history in the common ancestor of *Terrazoanthus* (node 13; Fig. 6) if either of the unnamed *Terrazoanthus* species included in the phylogeny has this character state. As with *E. illoricatus* and *P. cutressi*, it is unclear if the simplified mesogleal arrangement of *Terrazoanthus minutus* (Fig. 6) represents a state change or if the arrangement (known only from a drawing in the original description) represents an oversimplification of its true form.

A transition from cteniform endodermal to linear mesogleal marginal musculature, similar to extant *Palythoa*, occurred at the common ancestor of Brachycnemina (proportional likelihood = 0.5424; Figs. 3 and 7, node 14) and remained unchanged in the common ancestor of Sphenopidae (Fig. 7, node 15). A transition to orthogonally-reticulate mesogleal arrangement, similar to extant *Isaurus*, occurred at node 16 (proportional likelihood = 0.5160; Fig. 7) and preceded separate transitions to discontinuous mesogleal arrangement in the common ancestor of *Zoanthus* and *Acrozoanthus* (proportional likelihood = 0.9999; Fig. 7, node 17) and the autapomorphic discontinuous endodermal arrangement of *Neozoanthus caleyi* (Fig. 7).

### Functional morphology evolution

**Fig. 9:**
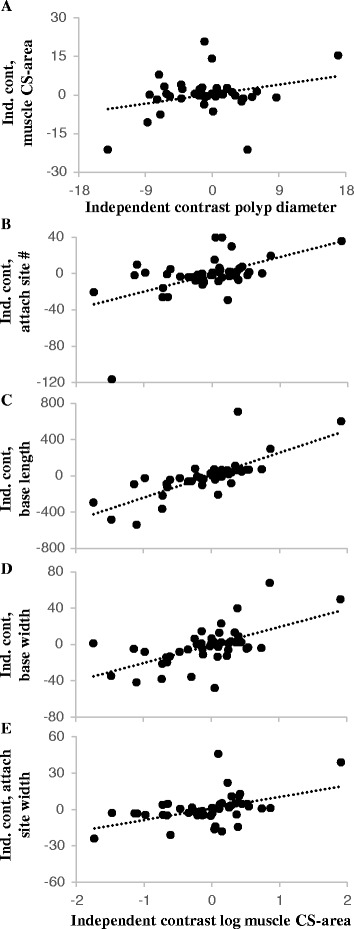
Multivariate regressions of size-corrected shape dimensions on size. Significant positive evolutionary allometry was detected between the size of polyps (diameter) and the cross-sectional area of the marginal musculature [r^2^ = 0.093, *p* < 0.049] (**a**), and the marginal musculature (cross-sectional area) and the number of muscle attachment sites [r^2^ = 0.286, *p* < 0.001] (**b**), length of the mesogleal base [r^2^ = 0.587, *p* < 0.001] (**c**), width of the mesogleal base [r^2^ = 0.363, *p* < 0.001] (**d**), and width of the attachment sites [r^2^ = 0.296, *p* < 0.001] (**e**)

The best-fit for each of the pair-wise size and shape character regression-residual sets mapped onto the composite phylogeny is the punctuated average with branch lengths model [36]; indicating that the data are phylogenetically structured (with likelihood scores 1–2 orders of magnitude larger than other phylogenetically structured models, and 2 orders of magnitude larger than a star phylogeny) and conserved (with daughter nodes commonly retaining the phenotypic state of the parent at each bifurcation) with phenotypic divergence consistent with the evolutionary rates of the molecular phylogeny (branch lengths are representative of phenotypic divergence). Because the regression-residuals of the phenotypic data are phylogenetically structured, phylogenetic regression is an appropriate test to assess covariance between characters. Using phylogenetic independent contrasts (PIC), we detected a significant pattern of associated variation between polyp and muscle size, and between functional components of the marginal musculature across the evolution of Zoanthidea (Fig. 8). As the size of polyps (diameter) increases, so does the (polyp) size-corrected cross-sectional area of the marginal musculature (*r*^2^ = 0.309, *p* < 0.041). As the log-transformed cross-sectional area of the muscle increases, so do the (muscle) size-corrected number of muscle attachment sites (Pearson’s *r* = 0.550, *p* < 0.001), length of the mesogleal base (*r* = 0.760, *p* < 0.001), width of the mesogleal base (*r* = 0.560, *p* < 0.001), and width of the attachment sites (*r* = 0.473, *p* < 0.001). Using the nodal contrast values from the PIC analysis in a simultaneous multivariate regression and permutation test indicates significant positive evolutionary allometry between the size of polyps (diameter) and the cross-sectional area of the marginal musculature (*r*^*2*^ = 0.093, *p* < 0.049; Fig. 9a), and the marginal musculature (cross-sectional area) and the number of muscle attachment sites (*r*^*2*^ = 0.286, *p* < 0.001; Fig. 9b), length of the mesogleal base (*r*^2^ = 0.587, *p* < 0.001; Fig. 9c), width of the mesogleal base (*r*^2^ = 0.363, *p* < 0.001; Fig. 9d), and width of the attachment sites (*r* = 0.296, *p* < 0.001; Fig. 9e).

## Discussion

### Diversity of marginal muscle form

Within the diversity of Zoanthidea taxa that we examined, we can report with considerable certainty that there are at least ten distinguishable categories of marginal musculature form (branchiform endodermal, cteniform endodermal, spindly-cteniform endodermal, discontiguous endodermal, meso-endo transitional, cyclically transitional, discontiguous mesogleal, linear mesogleal, reticulate mesogleal, and orthogonally-reticulate mesogleal), and with skepticism that there are two additional forms (simplified mesogleal and endo-meso transitional). That this study represents the minimal diversity of form is evident in the taxon sampling; although this phylogeny is by far the most comprehensive available (both in terms of taxa and molecular characters), it is limited to ~40 % of the known diversity of Zoanthidea species and the true diversity is likely to be much greater (as newly discovered taxa have recently and rapidly proliferated). Certainty in these analyses arises from direct examination of histological sections and comparisons with closely related taxa; skepticism arises from reliance on published drawings of histological sections (simplified mesogleal) and confusion surrounding the publication of original species identifications (misidentification of *Savalia savaglia* as *Antipathozoanthus macaronesicus*; see Swain & Swain [34] for a detailed discussion) and their similarity to known forms as they would be interpreted from a single histological section (endo-meso transitional; see Fig. 2).

The diversity of form identified here represents a significant (at least five-fold) expansion in our understanding of the diversity of marginal muscle forms in Zoanthidea. The traditional interpretations are partitioned and the breadth of possible arrangements expanded. The historical endodermal character state encompassed known variation that is designated here as branchiform endodermal, cteniform endodermal, and discontiguous endodermal forms (Fig. 1); the spindly-cteniform endodermal arrangement was first described as transitional [37], however further examination of sections in series revealed a pattern of attachment sites inconsistent with that interpretation [34]. The historical mesogleal character state encompassed known variation that is designated here as linear mesogleal, reticulate mesogleal, orthogonally-reticulate mesogleal, and (in part) meso-endo transitional forms (Fig. 1). The distinction between mesogleal and transitional forms were often blurred, as a proximal tail of mesogleal pleats following muscle fibers enclosed in lacunae was alternatively considered mesogleal or transitional. The newly designated meso-endo transitional form has a proximal tail of mesogleal pleats that accounts for approximately half the length of the muscle and lacunae organizations that are reminiscent of a section through the stacks of cisterna in all specimens examined (Fig. 6). The divided (or double) mesogleal form was historically considered a variation of the mesogleal form, but here is designated as a separate character state: discontiguous mesogleal (Fig. 1). The transitional form was historically considered a variation on the mesogleal (and sometimes endodermal) form that demonstrated the evolutionary development of the marginal musculature [23, 25] and encompassed cyclically transitional, (in part) meso-endo transitional, and (in part) mesogleal forms.

### Evolutionary relationships among extant forms

The data underlying the phylogenetic analyses originates from diverse sources (Additional file 1: Table S1 and Additional file 2), ranging from histology prepared from the same specimen as the DNA used to infer phylogeny (highly reliable match between form and phylogeny), to drawings and written descriptions culled from published species definitions paired with different specimens as the DNA source (leaving an opening for a mismatch between morphology and molecules); therefore the results must be interpreted within recognition of its limitations. Although imperfect, this is the extent of our current knowledge.

**Fig. 10:**
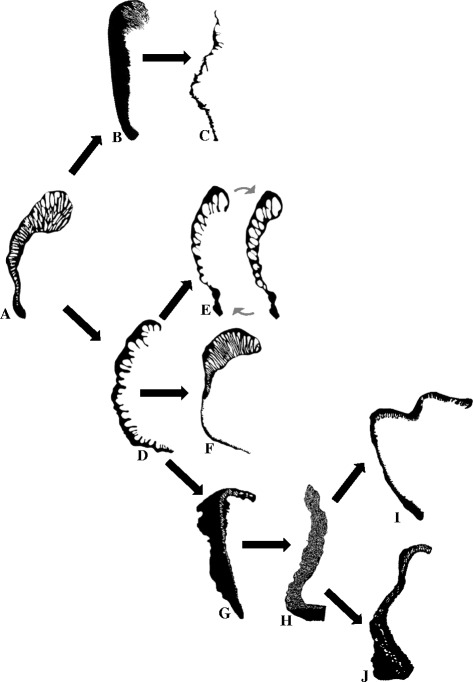
Summary of reconstructed transitions for marginal musculature form in the Zoanthidea. The marginal muscle forms are reticulate mesogleal (**a**), branchiform endodermal (**b**), spindly-cteniform endodermal (**c**), cteniform endodermal (**d**), cyclically transitional (**e**), meso-endo transitional (**f**), linear mesogleal (**g**), orthogonally-reticulate mesogleal (**h**), discontiguous endodermal (**i**), discontiguous mesogleal (**j**)

The major clades of Zoanthidea taxa that are identifiable through molecular (Fig. 3) or ecological characters [32] are also largely circumscribed by marginal muscle form (Figs. 4, 5, 6, and 7). For some genera, the marginal musculature appears to be unambiguously definitive (*Isozoanthus*, *Neozoanthus*, *Isaurus*, *Zoanthus*, and *Palythoa*), for others the differences in form are more subtle (*Microzoanthus, Epizoanthus, and Terrazoanthus*) or seem to span multiple genera (*Corallizoanthus* and *Savalia*, or *Antipathozoanthus*, *Parazoanthus*, and *Hydrozoanthus*). The spindly-cteniform endodermal arrangement of *Microzoanthus* could be confused with the cteniform endodermal arrangement (Fig. 3), save that the former has a sigmate mesogleal base and is generally more diminutive and sparsely populated by attachment sites (Fig. 1; Fig. 8). The reticulate mesogleal arrangement of *Epizoanthus* could be confused with the meso-endo transitional arrangement of *Terrazoanthus* (Figs. 4 and 6), as the former may include forms with a proximal tail of mesogleal pleats following muscle fibers enclosed in lacunae; however they are distinguishable by the shape and organization of the lacuna (irregular lacunae in a reticulate mesh compared to elliptical lacunae arranged in a stack). The presence of a proximal tail of mesogleal pleats appears to be a homoplasious feature of a few *Epizoanthus* species (*Epizoanthus cf. ramosus* and *Epizoanthus aff. arenaceus* [HI]; Fig. 4), while it is a definitive feature of *Terrazoanthus*. While these two pairs of forms (cteniform endodermal & spindly-cteniform endodermal, and reticulate mesogleal & meso-endo transitional) are morphologically similar (but not indistinguishable; Fig. 1), their similarity is derived through convergence (i.e. the morphological similarity between forms is not a shared derived character, but originates through convergent evolution of disparate linages) and are evolutionarily distinct (Figs. 8 and 10).

The cyclically transitional arrangement of *Corallizoanthus* and *Savalia* may actually be two distinct forms that are definitive for each genus: in *Corallizoanthus* the attachment sites transition in cycles between endoderm-facing pleats and mesogleal lacunae, and in *Savalia* the attachment sites transition in cycles between endoderm-facing pleats, ectoderm-facing pleats, and mesogleal lacunae (Figs. 2 and 5). The ectoderm-facing pleats and the complete transition through the mesoglea of *Savalia* appear to be unique among the Zoanthidea (and perhaps all of Anthozoa), and may be useful as a definitive feature of *Savalia* once additional species in this genus are thoroughly examined in serial section. The cteniform endodermal arrangement seems to span three genera (*Antipathozoanthus*, *Parazoanthus*, and *Hydrozoanthus*) and two families (Parazoanthidae and Hydrozoanthidae), however these taxa are distinguishable by other characters [34].

### Evolution of marginal muscle form

The ancestral state reconstruction recovered at least nine (the minimum number required to achieve ten character states), and as many as 15 transitions between muscle arrangement states. All of the transitions above the necessary minimum involve morphologies known only from drawings (simplified mesogleal arrangement of *E. illoricatus*, *P. cutressi*, and *T. minutus*) or histological sections that did not originate from the same specimen as the molecular data (*Antipathozoanthus macaronesicus, Savalia lucifica*). The extreme simplicity of form reported for the marginal musculature of *E. illoricatus*, *P. cutressi*, and *T. minutus* (which is entirely homoplasious and resulted in 3 terminal transitions; Figs. 4, 5, and 6) may be due to oversimplified interpretations by the original authors, an artifact of histology performed on species that are among the smallest known, or misidentified specimens. The morphology of *Savalia lucifica* (which resulted in the reconstruction of two independent origins of the cyclically transitional arrangement: Fig. 5, nodes 8 & 9) is quite certain as it was assessed using the holotype specimen (USNM 50975); however the molecular characters originated from a different specimen [32] which was not publically described nor vouchered and could therefore be misidentified [34]. The identity and attributes of *Antipathozoanthus macaronesicus* (which resulted in a transition and a reversal in *Antipathozoanthus hickmani*: Fig. 5, node 11) have been confused since the original description (and subsequent publication of DNA sequences) where specimens were misidentified [34]. If further research is able to show that these five taxa are actually more similar to their closest relatives than is currently demonstrable, the form of the marginal musculature may be largely synapomorphic and sufficiently reflective of evolution that its status as a key character in Zoanthidea systematics will be more fully restored.

The evolutionary origin and progression of the Zoanthidea marginal musculature has long been thought to mirror its embryonic development [19, 23, 25]. The first modern assessment of this hypothesis identified at least five transitions to the mesogleal form, but was not able to discern the ancestral state of the order using a reconstruction based on two character states (endodermal and mesogleal) and nearly the same phylogeny that is employed here [32]. Here we use much more detailed characterizations of the marginal musculature (at least ten states) and recover a reticulate mesogleal arrangement at the origin of Zoanthidea and a complex series of state transitions (Fig. 10) that are not consistent with the historical hypotheses. The reticulate mesogleal form is symplesiomorphic and ancestral to all other states, and is reconstructed to have transitioned directly to the branchiform endodermal and cteniform endodermal arrangements (Fig. 10). The spindly-cteniform endodermal arrangement may be derived from the branchiform endodermal form (Fig. 10), however inclusion of additional *Microzoanthus* and *Isozoanthus* species in the analysis would help to clarify the sequence of transitions in this region of the phylogeny. The cyclically transitional, meso-endo transitional, and linear mesogleal arrangements are all derived from the cteniform endodermal form (Fig. 10); transitions that would simply require partial or complete circumscription of the existing broadly-anchored endodermal muscles through expansion of the mesoglea. The orthogonally-reticulate mesogleal arrangement is derived from linear mesogleal, which is subsequently reconstructed to give rise to the discontiguous endodermal and discontiguous mesogleal forms (Fig. 10); however, data for the taxa in this region of the phylogeny are sufficiently sparse (*Isaurus* and *Neozoanthus*; Fig. 7) that additional taxa and data may alter this interpretation.

These conclusions generally agree with the analysis of Rodriguez et al. [28] about the earliest form of the Zoanthidea marginal musculature (however, the absence of Zoanthidea without marginal muscles does not support an origin of the hypertrophy within the order, but perhaps at its origin) and mirrors the progression of evolutionary transitions in Actiniaria; in both anthozoan orders, mesogleal forms of the marginal musculature are ancestral and endodermal forms are derived through convergent evolution.

### Functional morphology evolution

The evolution of polyp size requires compensatory phenotypic change in the marginal musculature to produce sufficient force to curl the margin of the column over the retracting oral disk and tentacles. Disparity between polyp diameter and muscle cross-sectional area (proportional to force) is both phylogenetically structured and conserved, indicating that closely related species require similar retraction mechanics. The muscle cross-sectional area is influenced by variation in polyp size, independent of phylogeny, where increasing polyp diameter has an allometric association with more robust muscles; suggesting that the largest muscles are required to deform the biggest polyps and create additional force than is necessary for minimal retraction.

As with other Anthozoa, the largest marginal muscles are often seen in shallow-water species (genera *Isaurus*, *Palythoa*, *Protopalythoa*, & *Zoanthus*) that can inhabit either intertidal or wave-swept areas [38] and the smallest marginal muscles are often seen in species that are infaunal (genus *Microzoanthus*; [37]) or can partially or entirely retract within host structures (genus *Parazoanthus*; [39, 40]). Disproportionally enlarged marginal muscles allow more forceful and complete retraction of the oral disk and tentacles to protect these structures from desiccation, abrasion, and predation inherent to near-surface habitats. However, there are notable exceptions that do not conform to this general hypothesis. *Acrozoanthus* have marginal muscles that rank among the smallest in CS-area (Fig. 8) and are known from intertidal mudflats (where desiccation would clearly be an issue) and shallow habitats symbiotically associated with tube-worms of the genus *Eunice* [41]. Similarly, *Epizoanthus illoricatus* (also a symbiont of Polychaeta [32]) has among the smallest marginal muscle CS-areas known (Fig. 8) and is unusually diminutive for an *Epizoanthus* species. Symbionts of Anthozoa, Hydrozoa, and Demospongiae [32] all have among the smallest marginal muscle CS-areas (Fig. 8) and may have limited retraction abilities that could be mitigated by their symbiotic associations (i.e. part of the benefit of symbiosis may be physical protection, especially among Demospongiae symbionts, that reduces the importance of complete retraction in these species). On the opposing extreme are *Isozoanthus*, which have some of the largest marginal muscle CS-areas (Fig. 8) and live unassociated at 20–100 m depth [32, 42]. The retraction mechanisms of these *Isozoanthus* species are curiously extravagant; not only are the marginal muscles exceptionally enlarged, but the longitudinal mesenteric retractor muscles of the directive mesenteries are so powerful that they allow the retracted column to be further inverted, completely covering the capitulum and resulting in a visible seam (rather than the typical puckered capitulum adorned with ridges corresponding to the underlying pairs of tentacles) along the directional axis at the distal apex ([32, 42]; Morphbank collections 477928 & 477929). These *Isozoanthus* species may face severe selection for protection of the oral disk and tentacles (in the absence of desiccation, perhaps predation) to have evolved such a powerful retraction mechanism that so dramatically contorts the column. Similarly, many of the free-living taxa [32], and those that are symbionts of Crustacea [32], have among the largest marginal muscle CS-areas known (Fig. 8). Perhaps living independent of potentially protective symbiotic hosts, or living on the shells of actively mobile hosts, present greater requirements for retraction.

The evolution of marginal muscle size, and therefore force generating potential (CS-area is proportional to force), requires compensatory phenotypic change in the mesogleal support structures to accommodate sufficient surface-area for attachment and support to transfer forces for distorting the mesoglea and contracting the polyp. Disparity between muscle size and scaffold shape is both phylogenetically structured and conserved, indicating that closely related species employ similarly composed retraction mechanisms. The morphology of the mesogleal support structures are highly influenced by variation in muscle size, independent of phylogeney, where increasing muscle size is allometrically associated with more robust and complex scaffolding; suggesting that robust support structures are required to resist the additional stress generated by the largest muscles.

Although it is intuitive that increased muscle size requires increased support structure robustness, it was not clear from the diversity of forms observed that this should be true of all shape parameters. In the elongated and broadly attached arrangements of the endodermal forms (Fig. 1a–h), similar support for increased muscle size could be accomplished through elongation alone, expansion of the basal mesoglea and attachment sites, or both. In the concentrated and circumscribed arrangements of the mesogleal forms (Fig. 1i–j, q–t), elongation of the proximal tail would result in minuscule gains in CS-area, but increased muscle size could be accommodated through expansion of all support structures of the concentrated distal head of the muscle. In the diffuse and circumscribed arrangements of the mesogleal forms (Fig. 1m–p), increased muscle size could only be accommodated through elongation of the base and expansion of the lacunae. It remains to be seen if a similar pattern exists in other anthozoan orders where the marginal musculature is independently derived, particularly the Actiniaria where some forms of the marginal musculature (e.g. pinnate and palmate endodermal arrangements) are not observed in the Zoanthidea and could accommodate increased muscle CS-area with very different compensatory changes in the mesogleal support structures (which can be quite arbuscular in the Actiniaria).

## Conclusions

Retraction mechanisms of extant Zoanthidea are more diverse, and their evolutionary histories more complex, than previously recognized. Historical dual-state characterization is both insufficient and homoplasious, with similar forms derived through convergent evolution; in an evolutionary pattern that is reminiscent of the Actiniaria. Multi-state characterization described here is largely reflective of evolution and may offer a key feature for integrative systematics within an order whose higher taxa lack definitive features because they have been described through molecular parataxonomy. Evolution of the marginal musculature and its support structures are not strictly scaled according to variation in size, but vary through evolutionary allometry in response to requirements for additional force and resistance.

## Methods

Extant marginal muscle forms of Zoanthidea were compiled, measured, and categorized by the arrangement of mesogleal attachment and support structures. Evolutionary patterns in the arrangement and form of the marginal musculature of extant taxa and their ancestors were revealed by mapping their features onto the most comprehensive molecular phylogeny available and examining their origin and evolutionary progression.

### Morphological data collection

Features of the marginal musculature and diameters of polyps were compiled from a review of original species descriptions, revisions, new histological sections, and histological sections prepared for Swain [32], Swain & Swain [34], and John Ryland of Swansea University, United Kingdom. Wherever possible, we examined the morphology of the same specimens that were used to construct the molecular phylogeny (25 of 60 taxa; See Additional file 1: Table S1 and Additional file 2). New histological sections were prepared following the protocols of Swain [33] and Swain & Swain [34] with specimens (USNM 50975, 1086480) sampled during a visit to the United States National Museum of Natural History, Washington D.C., USA.

The overall shape of the marginal musculature is challenging to quantify because the curvature of the mesoglea and exact shape of lacuna or pleats are dependent upon the degree of polyp constriction at the moment of preservation [43], particularly for specimens that may not have been prepared using identical methods or preservatives (such as is typical with museum collections where specimens are obtained under diverse circumstances, goals, and methods). All of the specimens used in this study were partially to fully retracted, we therefore declined to apply methods that allow quantification and comparison of the specific shape of mesogleal structures (e.g. Klingenberg & Gidaszewski [44]) in favor of repeatable binning into categories, counting repeated structures, and measuring general components of shape.

Images of the marginal musculature in longitudinal section (collected from histology and published photos, drawings, and descriptions) were standardized for comparison and analysis by creating new drawings depicting the columnar mesoglea of the marginal region with the muscle attachment sites. Marginal musculature arrangements (of 58 taxa; Additional file 1: Table S1 and Additional file 2) were categorized according to the location and morphology of the mesogleal structures that function as muscle attachment sites (Fig. 1). Features of the marginal muscles (mean base mesoglea length, mean maximum base mesoglea width, mean maximum attachment site width, mean muscle cross-sectional area, and mean attachment site count) were collected from images and drawings (of the 49 taxa for which scale could be determined; Additional file 1: Table S1 and Additional file 2). Considerable shrinking of tissues prepared for histology is expected and therefore the measurements of morphological features cannot be used to precisely estimate the size and shape of live organisms; however we are assuming the effect will be similar across taxa and that relative comparisons among taxa are valid. The source of each character state of every taxon included in this study is documented in the Additional file 1: Table S1 and Additional file 2. A character by taxon matrix was assembled from the discrete marginal musculature arrangements and continuous quantitative data on size-corrected features of mesogleal support structures (proportional to cross-sectional area of the muscle), log-transformed cross-sectional area of the muscle (also repeated as a size-corrected version; proportional to the diameter of the polyp), and diameter of the polyps. Previous characterizations of the marginal muscles used broad categories (diffuse or concentrated, “strong”), whereas careful measurements of the cross-sectional area provides a proportional indication of the forces that the muscle can potentially generate. All new images of histology (with the precise physical location of collected measurements) and drawings are publicly documented in MorphBank.

### Phylogenetic hypothesis construction

The phylogeny presented here is an expansion of the topology of Swain [32] and represents our best understanding of the evolutionary relationships and history of Zoanthidea. The main topology follows the 93-taxon phylogeny of Swain [32], which was inferred through a ML analysis of nucleotide sequence from the nuclear internal transcribed spacer (ITS) and 28S ribosomal genes, and the mitochondrial 12S and 16S ribosomal and cytochrome oxidase I (COI) genes. This topology was amended (following Swain & Swain [34]) by adding two *Terrazoanthus* species (based on extreme similarity among nucleotide sequences of 16S, COI, and ITS genes; these taxa are likely conspecific to *Terrazoanthus* species present in the original phylogeny [34]), a *Microzoanthus* species (following the COI-based phylogeny of Fujii & Reimer [37]) and a *Neozoanthus* species (following the 16S-based phylogeny of Reimer et al*.* [45]) resulting in a composite 97-taxon tree. Branch-lengths of appended taxa were set equal to their closest relatives. Taxon names were updated from those applied to the Swain [32] phylogeny (Additional file 3: Table S2) following the recommendations of Reimer & Fujii [46], Reimer et al. [47], Sinniger et al. [48], Sinniger et al. [49], and Swain & Swain [34]. With these changes, and the reassignment of *P. cutressi* proposed here, the genus-level taxonomy of Zoanthidea included in the phylogeny largely reflects molecular evolution.

### Phylogenetic analyses

Patterns of evolutionary change in the marginal musculature were assessed by mapping extant character states onto the composite phylogeny of Zoanthidea, followed by phylogenetic analysis with Mesquite 2.75 [50] and MorphoJ 1.06c [51]. Extant marginal musculature arrangements (discrete categories), size (muscle cross-sectional area and diameter of polyps), and shape parameters (length, maximum base mesoglea width, maximum attachment site width, and attachment site count) were traced over the amended ML phylogeny of Swain [32] and visualized with Evolview [52]. Evolutionary transitions were identified through ancestral state reconstruction of marginal musculature arrangements using the ML criterion and single-parameter Markov model (Mk1) in the Stoch-Char module [53] of Mesquite.

As species data may not be independent of evolutionarily relationships, phylogenetic comparative methods may be necessary to accurately assess interspecific character change [54]; however, inappropriately applying these methods to data that lack phylogenetic signal are likely to result in poor statistical performance [55]. Prior to applying phylogenetic comparative methods, the phylogeny was preemptively pruned to include only the 49 taxa for which morphological measurements could be collected, as to not inadvertently influence (with missing data) the outcome of the analyses. All figures presented here use the full 97-taxon tree to place the results in the most comprehensive phylogenetic context possible and to highlight the current state of knowledge. Phylogenetic signal was assessed (using a method analogous to evolutionary model-fitting of nucleotide sequence data for phylogenetic inference) by ML fitting of the observed pair-wise size and size-corrected shape character regression-residuals (to meet the assumptions of phylogenetic independent contrasts) and the composite phylogeny to nine models of continuous trait evolution (including star phylogeny, or no phylogenetic signal) using the Continuous-character Model Evaluation and Testing (CoMET) module [36] of Mesquite. This analysis assesses topological and chronological structure in the data, revealing both the presence (or lack thereof) and structure of phylogenetic signal.

Size and size-corrected shape character pairs (with phylogenetic signal in their regression-residuals) were further examined for phylogenetically-corrected correlation and evolutionary allometry to assess the evolutionary relationships among the functional components of the marginal musculature and the size of polyps. Because cross-sectional area of the muscle is proportional to the forces that it can generate, the shape of the mesoglea scaffolding must accommodate evolutionary changes in both size and force. Correlations between size (log-transformed cross-sectional area of the muscle or expanded polyp diameter) and each shape metric (size-corrected dimensions of mesogleal scaffolding features or size-corrected muscle cross-sectional area) were assessed using Phylogenetic Independent Contrasts (PIC) analysis within the Phylogenetic Diversity Analysis Package (PDAP) [56] of Mesquite to account for patterns of evolutionary relatedness among taxa [54] and generate the independent contrasts necessary to evaluate evolutionary allometry. A multivariate regression (through the origin) of each of the independent contrasts of shape on independent contrasts of size characterizes evolutionary allometry of each shape parameter and a permutation test (of 10,000 iterations) against the null hypothesis of independence between shape and size evaluates its statistical significance [57–59]. The combined regression and permutation tests were performed with MorphoJ.

### Availability of supporting data

The data sets supporting the results of this article are available in Morphbank, publication collection 851144 at www.morphbank.net, and are included within this article and its additional files.

## Additional files

Additional file 1: Table S1. Individual character states and their sources. Taxon X character matrix that identifies individual data sources. Additional file 2:
**References and caption for Table S1.**
Additional file 3: Table S2. Shifting taxon binomials. Current usage of taxon binomials matched with their equivalent binomials used in the original phylogeny.
