# Supplementary material for: Evolution of anthozoan polyp retraction mechanisms: convergent functional morphology and evolutionary allometry of the marginal musculature in order Zoanthidea (Cnidaria: Anthozoa: Hexacorallia)
Source: BMC Evol Biol. 2015 Jun 30;15:123. doi: 10.1186/s12862-015-0406-1 (PMC4486433; doi:10.1186/s12862-015-0406-1)
Supplement: Additional file 2: — References and caption for Table S1. [file 12862_2015_406_MOESM2_ESM.docx]

**Evolution of anthozoan polyp retraction mechanisms: convergent functional morphology and evolutionary allometry of the marginal musculature in order Zoanthidea (Cnidaria: Anthozoa: Hexacorallia)**

Timothy D. Swain^1,2,5^, Jennifer L. Schellinger^3^, Anna M. Strimaitis^3^, and Kim E. Reuter^4^

**Additional references for Table A1.**

60. Carlgren OH. South African Actiniaria and Zoantharia. K Svenska Vetenskapsakad Handl. 1938;17(3):1–148.

61. Carlgren OH. Ceriantharia und Zoantharia der deutschen Tiefsee-Expedition. Dt Tiefsee-Exped*.* 1923;29(7):243–337.

62. Tischbierek H. Zoanthiden auf wurmröhren. Zool Anz. 1930;91:91–5.

63. Lwowsky F. Revision der gattung *Sidisia* Gray (*Epizoanthus* auct.). Zool Jahrb Abt Syst Geogr Biol Tiere. 1913;34:557–614.

64. Herberts C. Étude systématique de quelques zoanthaires tempérés et tropicaux. Tethys. 1972;S3:69–156.

65. Pax F: Die Korallenfauna der Adria. Teil 1: Krustenanemonen. Thalassia. 1937;2:3–66.

66. Sinniger F, Haussermann V. Zoanthids (Cnidaria: Hexacorallia: Zoantharia) from shallow waters of the southern Chilean fjord region, with descriptions of a new genus and two new species. Org Divers Evol. 2009;9(1):23–36.

67. Carlgren O. Zoantharia. Danish Ingolf-Exped. 1913;5(4):1–62.

68. Wood RL. Identification and microanatomical study of a new species of *Epizoanthus* (Zoanthidea). PhD Dissertation. University of Washington; 1957.

69. Reimer JD, Nonaka M, Sinniger F, Iwase F. Morphological and molecular characterization of a new genus and new species of parazoanthid (Anthozoa: Hexacorallia: Zoantharia) associated with Japanese Red Coral. Coral Reefs. 2008;27(4):935–49.

70. Cutress CE, Pequegnat WE. Three new species of Zoantharia from California. Pac Sci. 1960;14(2):89–100.

71. Ocaña O, Brito A. A review of Gerardiidae (anthozoa: Zoantharia) from the Macronesian Islands and the Mediterranean Sea with the description of a new species. Rev Acad Canar Cienc. 2003;15(3-4):159–89.

72. Reimer JD, Irei Y, Fujii T. Two new species of *Neozoanthus* (Cnidaria, Hexacorallia, Zoantharia) from the Pacific. Zookeys. 2012;246:69–87.

73. Pax F. Studien an westindischen Actinien. Zool Jahrb. 1910;S11(2):157–330.

74. Reimer JD, Ono S, Iwama A, Takishita K, Tsukahara J, Maruyama T. Morphological and molecular revision of *Zoanthus* (Anthozoa: Hexacorallia) from southwestern Japan, with descriptions of two new species. Zool Sci. 2006;23:261–275.

75. Duerden JE. Jamaican Actiniaria. Part I-Zoantheae. Sci Trans Roy Dub Soc. 1898;6:329–84.

76. Carlgren O. Ostafrikanische Actinien. Gesammelt von Herrn Dr. F. Stuhlmann 1888 und 1889. Mitt Naturh Mus. 1900;17:21–144.

77. Pax F, Mueller I. Zoantharien aus Viet-Nam. Mem Mus Natl Hist Nat (Paris). 1957;16:1–40.

78. McMurrich JP. The Actiniaria of the Bahama Islands, W.I. J Morph. 1889;3:1–80.

79. Ryland JS, Lancaster JE. Revision of methods for separating species of *Protopalythoa* (Hexacorallia: Zoanthidea) in the tropical West Pacific. Invertebr Syst. 2003;17:407–28.

80. Studer T. Übersicht der Anthozoa Alcyonaria, welche während der Reise S.M.S. Gazelle um die Erde gesammelt wurden. Monat Akad Wiss (Berlin). 1879;1878:632–88.

81. Walsh GE, Bowers RL. A review of Hawaiian zoanthids with descriptions of three new species. Zool J Linn Soc. 1971;50:161–80.

**Additional Table Captions**

**Table S1.** Individual character states and their sources. Citations are numbered, measurements collected from histological sections documented in Morphbank are denoted as “MB”, polyp diameters estimated from the contracted state are denoted as “estimated” (based on an average of 42% reduction in contracted polyp diameter in other taxa).
