# Supplementary material for: Evolution of anthozoan polyp retraction mechanisms: convergent functional morphology and evolutionary allometry of the marginal musculature in order Zoanthidea (Cnidaria: Anthozoa: Hexacorallia)
Source: BMC Evol Biol. 2015 Jun 30;15:123. doi: 10.1186/s12862-015-0406-1 (PMC4486433; doi:10.1186/s12862-015-0406-1)
Supplement: Additional file 3: Table S2. — Shifting taxon binomials. Current usage of taxon binomials matched with their equivalent binomials used in the original phylogeny. [file 12862_2015_406_MOESM3_ESM.docx]

| **Taxon binomen used here** | **Taxon binomen used by**  **Swain 2010** | **Unique ID** | **Citation** |
| --- | --- | --- | --- |
| *Corallizoanthus aff. tsukaharai* [NZ] | *Epizoanthus aff.* *tsukaharai* [NZ] | NZ 66 | 34 |
| *Corallizoanthus aff. tsukaharai* [CA] | *Epizoanthus aff.* *tsukaharai* [CA] | NMNH 258 | 34 |
| *Savalia lucifica* | *Parazoanthus lucificum* | SAV 3 | 48 |
| *Savalia savaglia* | *Gerardia savaglia* | SAV 1 | 48 |
| *Savalia savaglia* [Can Is] | *Gerardia macaronesica* | Smac | 34 |
| *Antipathozoanthus macaronesica* [CV] | Parazoanthid sp. [Cape Verde] | CV | 34 |
| *Antipathozoanthus macaronesica* [Pri] | Parazoanthid sp. [Principe] | PRI | 34 |
| *Antipathozoanthus hickmani* | Parazoanthid sp. [G1] | GAL 1 | 46 |
| *Parazoanthus cutressi* | *Epizoanthus cutressi* | TOB 44 | Proposed here |
| *Parazoanthus darwini* | *Parazoanthus aff. swiftii* [GAL] | GAL 2 | 46 |
| *Terrazoanthus* sp. [302] | Zoanthidea sp. [302] | S302 | 46 |
| *Terrazoanthus* sp. [YP] | Parazoanthid sp. [yellow polyp] | YP | 46 |
| *Terrazoanthus minutus* | *Epizoanthus minutus* | GM 3 | 34 |
| *Terrazoanthus patagonichus* | *Epizoanthus patagonichus* | PER 237 | 34 |
| *Terrazoanthus californicus* | *Epizoanthus californicus* | PER 243 | 34 |
| *Hydrozoanthus aff. tunicans* [Sul] | *Parazoanthus aff. tunicans* [SUL] | SUL 1 | 49 |
| *Hydrozoanthus tunicans* | *Parazoanthus tunicans* | TOB 40 | 49 |
| *Hydrozoanthus antumbrosus* | *Isozoanthus antumbrosus* | PAN 21 | 49 |
| *Hydrozoanthus cf. gracilis* [NC1] | *Parazoanthus cf. gracilis* [NC1] | NC 1 | 49 |
| *Hydrozoanthus gracilis* | *Parazoanthus gracilis* | NIP 153 | 49 |
| *Hydrozoanthus cf. gracilis* [NC2] | *Parazoanthus cf. gracilis* [NC2] | NC 2 | 49 |
| *Acrozoanthus australiae* [Indo] | *Acrozoanthus* sp. [FS-2005] | Acro05 | 47 |
| *Acrozoanthus australiae* [Sul] | *Acrozoanthus* sp. [Sulawesi] | Sul05 | 47 |

**Table S2**. Shifting taxon binomials
